# On Photographing Artists’ Books

**DOI:** 10.1007/s10912-019-09593-7

**Published:** 2019-12-20

**Authors:** Egidija Čiricaitė

**Affiliations:** Slade School of Fine Art, University College London, WC1E 6BT, London, UK

**Keywords:** Artists’ books, Photographing artists’ books, Artists’ publications, Photography, Documentation

## Abstract

Artists’ books are challenging to photograph. They function as a unit of tightly conceptually-bound visual, textual and material elements in addition to a heightened self-awareness of the work's booksness. Binding, size, weight, and shape of the book, translucency, texture, thickness of paper, placement of images and/or text on the page or off the page interact with other graphic elements; they control, and direct the reader towards the expressive components of meaning which arise from pace, haptic experience, and visual or structural stylistic choices. Most of such information gets sacrificed in the process of documentation. Here I discuss some of such issues of photographing artists books for this journal and my solutions to replicating each artists’ book within the physical and thematic constraints of this publication: I tried not only to visualize the books’ content but also to translate some of the experience of how that content makes itself meaningful to the reader.

The online version contains supplementary material available at 10.1007/s10912-019-09593-7.

Artists’ books offer a compelling minefield of issues for anyone trying to document them through photography. Not only do they bring a fascinating array of problems that arise from attempts to translate a three-dimensional haptic artwork into two dimensions, but they also have an added difficulty of being a time-based art medium. Indeed, artists’ books cannot be approached as content alone: they function as a whole of tightly conceptually-bound visual, textual and material elements in addition to a heightened self-awareness of the work’s *bookness*.

Photographing artists’ books reduces them to a flat image, stripping books of those qualities of objecthood and time, - that is, the *bookness* of the book. Structural elements (such as a type of binding), size, weight and shape of the book, translucency, texture, thickness of paper, placement of images and/or text on the page or off the page interact with other graphic elements; they control, and direct the reader towards the expressive components of meaning which arise from pace, haptic experience, and visual or structural stylistic choices. Most of such information gets sacrificed in the process of photographic documentation. However, the scale and nature of those sacrifices can be mitigated or adjusted based on the priorities of a project. What is the primary goal of the documentation? Is it to reproduce visual aspects of print? Is it to explain the process of making? Or is it to simulate the idea of the book to the best of possibilities?

In this special issue, our goal was to replicate each artists' book within the physical and thematic constraints of this journal, aiming not only to visualize the books’ content but also to translate some of the experience of *how* that content makes itself meaningful to the reader. As an academic publication printed in black and white in a preset standard paper and size, the *Journal of Medical Humanities* presented a fascinating challenge.

Although print color and format of the journal were non-negotiable, it was possible to salvage aspects of the haptic experience. In order to acknowledge the physical qualities of the artists’ books, I decided to integrate the existing journal structure as a framework for imitating book structures of the artists’ works: each one page of an artists’ book (whenever the book is bound on the spine) is superimposed onto one page of the journal, so to recreate the experience of the reader turning pages of the artists’ books. In addition, rather than replicating page spreads, the books were photographed as three-dimensional objects true to their actual size (where possible): our photographs are clearly showing reproductions of material bindings, instead of flat illustrations of drawings or poetry.

Not all five books chosen for the special issue are bound at the spine, nor do they all neatly fit into the size of the journal’s page. Yvonne J. Foster’s *Inside. An artist’s work: Living with Depression* was the easiest book to reproduce. It is published as a relatively small black and white pamphlet. I photographed the book from the top, keeping it open with the support of clips, erasers, rolls of washi tape and other small stationary objects. This book – like other books here – is printed on white paper and it contains large areas of empty white background around texts and images. When placed on a white surface it disappears into it. The lack of contrast between the book and the surroundings erases its outline. As a result, I decided to photograph the book against a sheet of matte black paper. Such choice not only improved the object’s visibility by framing it in a contrasting border, but also structured the special issue visually by clearly separating those pages containing images of artists’ books from the rest of the publication (mostly text-based content)

Bernard Fairhurst’s *Humpty Dumpty’s Bones* provides a different set of issues. The book is also bound at the spine, yet its size exceeds the page size of the *Journal of Medical Humanities*. In addition, *Humpty Dumpty’s Bones* includes a large number of semi-translucent pages overprinted with doctor’s comments, overlaying images of spine scans. I photographed the book's spreads “in motion” to suggest an impression of the turning page and to record the defusing effect of the transparencies. The coil binding of the book is centered on the gutter of the journal. The book was reproduced to the largest possible size.

Mary Rouncefield’s *Mr Darcy’s Advice to the Hip Patient* is a codex yet with special challenges of its own. Not only is the book too big for the journal’s pages, but it is also bound as an album and relies on an (almost) ritualistic turning of heavy sheets of paper hand-painted in luminous watercolors. There is a manuscript quality to *Mr Darcy’s Advice to the Hip Patient*, and this quality is, unfortunately, entirely lost in the process of reproduction. Although this artists' book is photographed as an object, the reader of the journal will only get to enjoy the book for its visual content in black and white. The vibrant colors of *Mr Darcy’s Advice to the Hip Patient* can be experienced in the online edition of the journal.

Pauline Lamont-Fisher’s *Dependency* and Anne Parfitt’s *Diary of an Illness* are both concertina (or accordion) books: as such, their folded structure cannot be reproduced within the constraints of an academic journal. *Dependency* is the smaller book of the two. It is printed on fine Japanese paper. The book traces a man's life journey, which ends in confusion and entaglement of Alzheimer's disease. The book is light and almost ethereal: such qualities inevitably get lost in photographic reproduction. *Dependency* includes images, stitching, and it is double-sided. For the journal, I have slightly shrunk the size of the images, so to display both sides of the concertina simultaneously allowing readers to be aware of the parallel progression of events in the book. *Dependency* was photographed as an open spread, with structure and folds clearly visible. *Diary of an Illness,* on the other hand, was photographed from the top as a volume of folded pages. The sheer size of the book makes it impossible to reproduce it page by page in the journal. *Diary of an Illness* is constructed of a seemingly infinite expanse of pages and bottles, representing the dazzling amount of medication that a person has to consume during cancer treatment. Photographing innumerable folded pages of the book recreates some sense of the work’s scale.

Artists’ books are challenging to reproduce for print. They are an integrated structure of numerous planes of material and meaning held together (or not) into a sequence activated by the reader, and, as a result, they do not lend themselves well to being reduced to a two-dimensional picture. While reproduction can salvage some aspects of experience, an artist’s book remains a multimodal form of artistic expression and it can only be truly enjoyed when handling the original object. The books presented in this special issue offer a meaningful alternative to the real works in the context of this journal publication. When possible, however, do have a look at those books in the flesh!

## Electronic supplementary material

ESM 1 (ZIP file 190 MB)

